# Stevens-Johnson Syndrome (SJS) As the Initial Presentation in a Patient With a New Diagnosis of HIV

**DOI:** 10.7759/cureus.50277

**Published:** 2023-12-10

**Authors:** Mollie Lagrew, Kelly L Perryman, Addie Walker, Paulette Hahn

**Affiliations:** 1 Ophthalmology, University of Florida, Gainesville, USA; 2 Internal Medicine, University of Florida, Gainesville, USA; 3 Dermatology, University of Florida, Gainesville, USA; 4 Rheumatology, University of Florida, Gainesville, USA

**Keywords:** toxic epidermal necrolysis (ten), ten, dermatology, hiv incidence, stevens-johnson syndrome (sjs)

## Abstract

A woman in her 40s presented to the emergency department with a diffuse rash consistent with Stevens-Johnson syndrome (SJS). There was no identifiable inciting factor. However, she was newly diagnosed with human immunodeficiency virus (HIV) during that same hospital admission. The leading theory for why she developed SJS given her lack of classic precipitating factors is an immune dysregulation as a result of HIV. Most cases of SJS/toxic epidermal necrolysis (TEN) in patients with HIV are related to highly active antiretroviral therapy and prophylaxis with trimethoprim-sulfamethoxazole. There is a lack of literature regarding SJS as the initial presentation of HIV without known underlying etiology or inciting factors.

## Introduction

Stevens-Johnson syndrome (SJS) and toxic epidermal necrolysis (TEN) are mucocutaneous reactions that are rare and potentially fatal. The annual incidence of SJS and TEN is estimated to be between two and seven per one million per year, with SJS outnumbering TEN three to one [1-4]. SJS and TEN are most commonly precipitated by medications. However, in patients with HIV, the incidence increases to 1 per 1000 [5]. It is important to recognize SJS and TEN, as they are associated with significant morbidity and mortality [6].

SJS and TEN are distinguished based on the extent of the body surface area involved. SJS is the least severe and is diagnosed when there is less than 10% skin detachment. A majority of patients will also have mucous membranes affected [7]. As previously mentioned, medications are the most likely causative factor and the medication in question should be promptly discontinued. An inciting factor is not always identified and in patients with new SJS, it is important to test for HIV, as the incidence of SJS is significantly higher in that specific patient population [5].

We report a case of a patient with SJS and a new diagnosis of HIV without medications as an inciting factor.

## Case presentation

An African American woman in her 40s presented to the emergency department with a three-day history of a diffuse rash. It began with itching of her chest and back and then became painful, spreading to involve her whole body including her palms and soles. She denied any new medications, supplements, or recent illnesses. Her medical history included hypertension, which was well-controlled on amlodipine.

**Figure 1:**
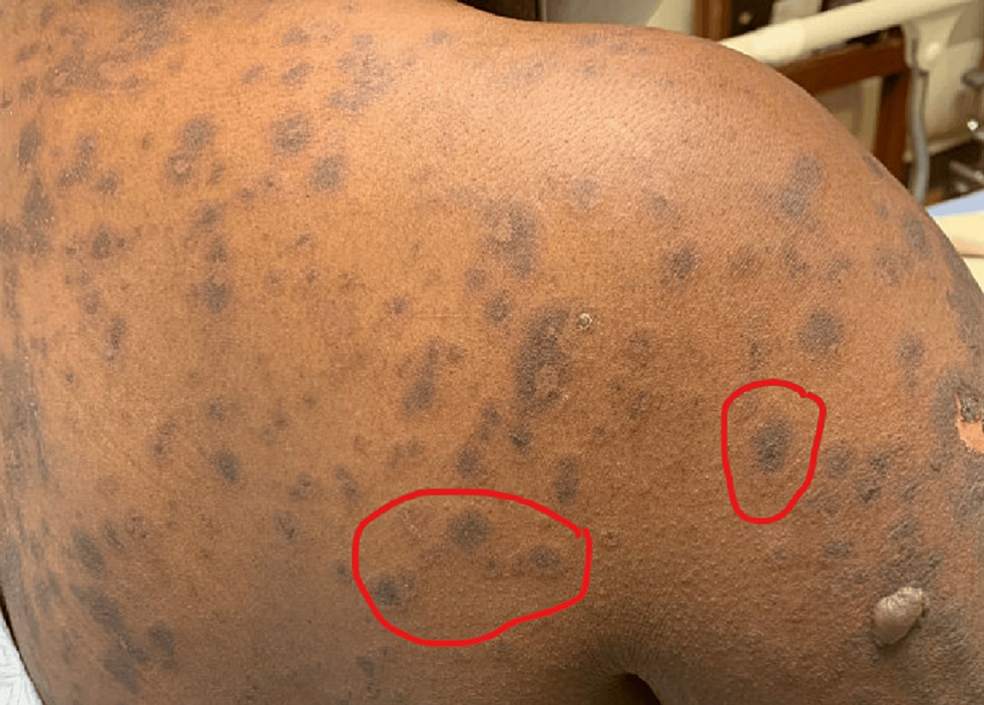
Atypical targetoid lesions demonstrating two zones of color (inner darker zone and outer lighter/slightly pink zone).

Upon presentation, she was afebrile and hemodynamically stable. Physical examination demonstrated diffuse ill-defined dusky patches with early bullae formation on the chest, back, upper arms, and abdomen involving less than 10% of her body surface area. There were few erosions on the chest and upper arms and atypical targetoid lesions with two zones of color and poorly defined borders of the upper flanks and posterior shoulders (Figure 1). Nikolsky was negative throughout. There was no mucosal involvement.

**Figure 2:**
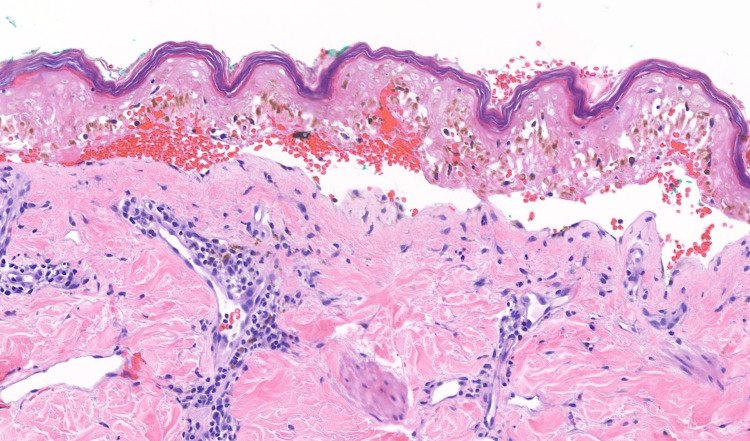
Punch biopsy of the lesion on the patient’s right arm, H&E 200x The epidermis is seen with full-thickness necrosis, orthokeratosis, and subepidermal cleft formation overlying the dermis with minimal perivascular chronic inflammation, consistent with the clinical impression of SJS. H&E: hematoxylin and eosin

Dermatology was consulted, and they performed a punch biopsy. Biopsy results demonstrated a basket weave stratum corneum overlying full thickness epidermal necrosis with focal subepidermal clefting and minimal dermal inflammation (Figure 2). The autoimmune panel revealed speckled antinuclear antibodies (ANA) with a titer of 1:320 but was otherwise negative. Infectious disease workup revealed elevated Chlamydia pneumoniae IgG titers (1:256), Chlamydia psittaci immunoglobulin G (IgG) titers (1:512), and an elevated Mycoplasma pneumoniae IgG, which likely resembled prior resolved infections. The syphilis screen was negative. The fourth-generation HIV antigen/antibody combo assay was repeatedly reactive and a new diagnosis of HIV was made. SJS was the leading diagnosis at presentation, given physical examination findings; however, no inciting event was obvious.

The patient was treated with cyclosporine 100 mg three times per day and gabapentin 300 mg three times per day for the pain. With the initiation of cyclosporine, there were no further bullae eruptions and the patient’s exanthem began to regress. Her pain was well-controlled with gabapentin. She remained afebrile and hemodynamically stable throughout admission. The patient was discharged on a cyclosporine taper and with a dermatology follow-up. She was referred to the health department to initiate treatment for HIV.

## Discussion

According to the World Health Organization, 1.5 million people were newly infected with HIV in 2020 with a reported 37.7 million people living with HIV worldwide [8]. The initial presentation of HIV ranges from asymptomatic to acutely ill. The skin is one of the more commonly affected organs in HIV and drug reactions are more prevalent in patients with HIV than in the general population [9]. Due to this potential spectrum of disease presentation in a patient presenting with SJS/TEN, it is important to consider and test for HIV.

SJS and TEN are distinguished based on the extent of the body surface area involved. SJS is the least severe and is diagnosed when there is less than 10% skin detachment. A majority of patients will also have mucous membranes affected [7]. The diagnosis can be made with a thorough clinical history, physical examination, laboratory workup, and biopsy. Clinical history should include medications and recent illness. A physical examination will likely reveal a painful erythematous rash ranging from erythematous macules, targetoid lesions, or diffuse erythema progressing to vesicles and bullae that are Nikolsky sign positive. However, in patients with darker skin tones, the lack of erythema does not preclude SJS. The rash of erythema multiforme also involves less than 10% of the body's surface area. It must include targetoid lesions and may include atypical targetoid lesions. As differences between typical and atypical targetoid lesions can be subtle, dermatology consultation is recommended in these cases. It is important to be aware that EM and SJS show identical histologic features on biopsy, and the dermatologist’s clinical impression is essential for distinction. In this case, the lack of typical targetoid lesions excluded EM from the clinical differential.

There have been multiple reports of SJS/TEN in patients with HIV who are prescribed nevirapine and/or trimethoprim-sulfamethoxazole [10-12]. As previously discussed, medications are the most common inciting factor. However, there is a lack of data in the literature when considering a patient presenting with SJS and a new HIV infection in the absence of Nevirapine and/or trimethoprim-sulfamethoxazole use.

The most popular theories regarding the hundred-fold increase in the incidence of SJS in patients with HIV are exposure to medications such as nevirapine and trimethoprim-sulfamethoxazole, immune dysregulation, and concomitant infections [9,13]. In our patient, immune dysregulation is the leading theory due to her lack of medication exposure and otherwise negative infectious disease workup. In her treatment future, her history of SJS should be considered when prescribing highly active antiretroviral therapy (HAART) and prophylaxis if indicated.

## Conclusions

When SJS/TEN is reported in the literature in patients with HIV, it is most associated with nevirapine and trimethoprim-sulfamethoxazole. However, there is a lack of reported data regarding a patient presenting with SJS and a new diagnosis of HIV in the absence of any other inciting factors. Therefore, it is important for the clinician to consider and test for HIV in a patient presenting with a new diffuse rash. The leading theory for why this patient developed SJS is immune dysregulation as a result of HIV infection. She responded well to cyclosporine, and HIV treatment was initiated early and efficiently. In her treatment future, her history of SJS should be considered when prescribing HAART and prophylaxis if indicated. 
